# Refractory ventricular fibrillation secondary to hyperkalemia resuscitated with extracorporeal membrane oxygenation: A case report

**DOI:** 10.1016/j.heliyon.2024.e31178

**Published:** 2024-05-13

**Authors:** Kun-Te Lin, Fu-Yuan Siao

**Affiliations:** a Department of Emergency and Critical Care Medicine, Changhua Christian Hospital, Changhua, 500, Taiwan; b Department of Kinesiology, Health and Leisure, Chienkuo Technology University, Changhua, 500, Taiwan; c Department of Mechanical Engineering, Chung Yuan Christian University, Taoyuan, 320, Taiwan

**Keywords:** Ventricular fibrillation, Hyperkalemia, Extracorporeal membrane oxygenation

## Abstract

The routine use of extracorporeal cardiopulmonary resuscitation (ECPR) is not recommended for patients with cardiac arrest. However, ECPR is considered for selected patients with cardiac arrest of reversible cause. Extracorporeal membrane oxygenation (ECMO) provides temporary cardiopulmonary support and adequate perfusion to the end organs, thereby shortening ischemic organ time and minimizing complications. One indication for ECPR therapy is prolonged ventricular fibrillation despite optimal conventional CPR.

Here, we report a successful recovery case from ECPR, in which the patient suffered from refractory ventricular fibrillation and was predisposed to severe hyperkalemia. Ventricular fibrillation failed to respond despite prolonged conventional CPR and defibrillation management for 32 min. After successfully initiating ECPR 54 min after cardiac arrest, spontaneous circulation returned sooner. He demonstrated clear consciousness after treatment and was discharged without any neurological disability on day 11.

## 1 Introduction

Extracorporeal cardiopulmonary resuscitation (ECPR) is increasingly used for patients with cardiac arrest for whom conventional CPR does not restore spontaneous circulation. Extracorporeal membrane oxygenation (ECMO) provides circulation, gas exchange, and organ perfusion while the reversible cause of cardiac arrest can be identified and treated [1,2]. Close monitoring of coagulation, hemostasis, and bleeding events should be maintained after adapting venoarterial extracorporeal membrane oxygenation (VA-ECMO) support [3].

Hyperkalemia is an emergency, with nonspecific initial symptoms. Electrocardiography (ECG) is important for detecting arrhythmia and hyperkalemia manifestations such as peaked T wave, flattened P wave, prolonged PR interval, widened QRS wave, and bradyarrhythmias. Hyperkalemia should be treated with calcium for maintaining cardiac function, insulin and sodium bicarbonate for the transcellular shift of potassium into cells, diuretic for lowering potassium, and hemodialysis for eliminating potassium in patients with impaired renal function or arrhythmias. ECMO is suitable for restoring circulation and providing bridge therapy before potassium elimination in patients experiencing cardiac arrest due to hyperkalemia [4].

## 2 Case presentation

**Fig. 1:**
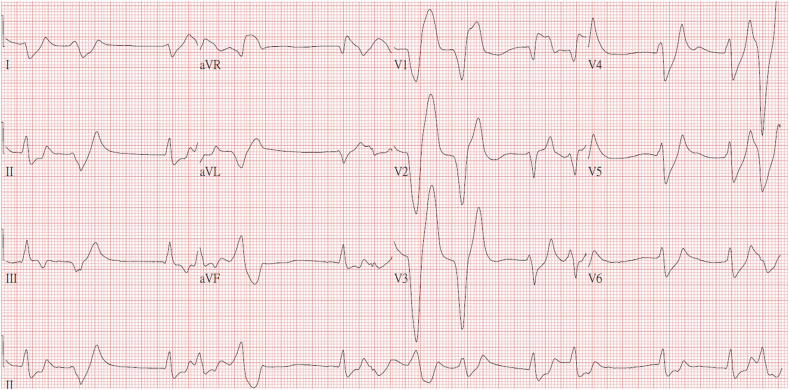
Electrocardiogram upon arrival at the emergency department.

A 45-year-old man with a diabetes mellitus history presented with a change in consciousness and hypotension in the emergency department (ED). Initially, his ECG revealed arrhythmia with a wide QRS complex and a peaked T wave (Fig. 1). The rapid dextrostix test revealed hyperglycemia. He received treatment according to his ECG presentation, including 400 mg of calcium chloride, 10 units of regular insulin, and 51 mEq of sodium bicarbonate injections, under the impression of hyperkalemia. However, sudden ventricular fibrillation occurred 5 min after arrival at ED. Consequently, CPR, defibrillation, epinephrine injection, and intubation with oxygen support were initiated. Blood sampling revealed severe hyperkalemia (9.2 mmol/L), metabolic acidosis, and hyperglycemia (Table 1). He was diagnosed with cardiac arrest with ventricular fibrillation secondary to severe hyperkalemia. Refractory ventricular fibrillation persisted for 32 min under resuscitation despite the administration of total calcium chloride of 800 mg, regular insulin of 30 units, and sodium bicarbonate of 170 mEq injection for extreme hyperkalemia management. We introduced VA-ECMO support for ECPR as salvage resuscitation management. The initial ECMO settings were a pump speed of 2800 revolutions per minute and a blood flow of 3.5 L/min. Heparin was administered as an anticoagulant for the use of ECMO. The return of spontaneous circulation (ROSC) was achieved sooner after ECMO rescue. ECMO treatment was initiated 54 min after cardiac arrest.

**Table 1:** Laboratory data in the emergency department.

Complete blood count			Arterial blood gas		
WBC	16.1 × 10^3^	/μL	pH	6.693	
Hb	12.2	g/dL	PCO_2_	24.0	mmHg
RBC	3.83 × 10^6^	/μL	PO_2_	287.0	mmHg
Platelet	304 × 10^3^	/μL	HCO_3_^−^	2.9	mmol/L
Neutrophil	89.3	%	O_2_ saturation	99.3	%
Lymphocyte	4.7	%	Base Excess	−33.1	mmol/L
**Biochemical examination**					
Glucose	1564		mg/dL		
Na	110		mmol/L		
K	9.2		mmol/L		
BUN	59		mg/dL		
Creatinine	2.92		mg/dL		
hs-Troponin-I	27.6		ng/L		

Note: WBC: white blood cells; Hb: hemoglobin; RBC: red blood cells.

**Fig. 2:**
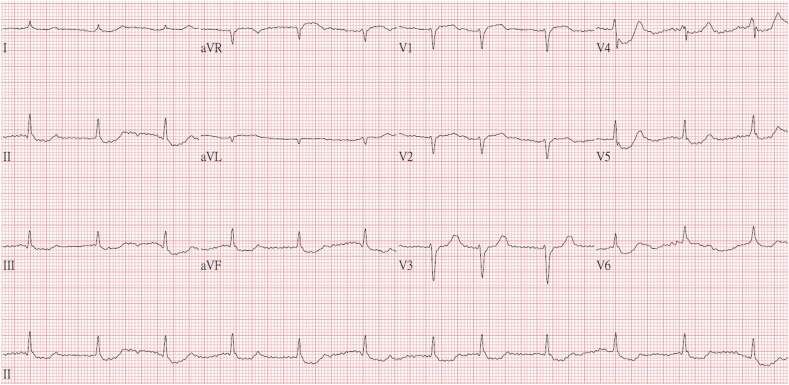
Electrocardiogram after extracorporeal membrane oxygenation.

Follow-up laboratory data revealed improvement in hyperkalemia (5.9 mmol/L) and metabolic acidosis (Table 2). No severe hypoxemia was observed after ECMO support. No wide QRS complex or peaked T wave was observed on the follow-up ECG examination (Fig. 2). Continuous venovenous hemofiltration (CVVH) was initiated for potassium excretion as part of the ongoing management. Furthermore, the patient received mechanical ventilation support, norepinephrine and vasopressin to constrict blood vessels, and insulin to lower blood glucose in the intensive care unit (ICU) (Table 3). Target temperature management (TTM) was not administered to the patient because his temperature was lower than 36°C after ROSC. The patient was successfully weaned off ECMO 3 days after ICU admission. His general condition improved, vital signs were stable without vasopressor administration, and hyperglycemia and severe diabetic ketoacidosis were corrected. He was extubated 6 days after admission. His consciousness gradually improved, and he was discharged 11 days after admission without further complications or neurological deficits. His modified Rankin scale score was 0 after discharge.

**Table 2:** Laboratory data after extracorporeal membrane oxygenation initiation.

Biochemical examination			Arterial blood gas		
K	5.9	mmol/L	pH	6.91	
Β-hydroxybutyrate	>7.0	mmol/L	PCO_2_	23.5	mmHg
			PO_2_	80.8	mmHg
			HCO_3_^−^	4.7	mmol/L
			O_2_ saturation	92.1	%
			Base Excess	−26.8	mmol/L

**Table 3:** Initial management in the intensive care unit.

Mechanical ventilator setting		Medication	
Mode	P-ACV	Norepinephrine	0.8 mg/h
I/E:	1.0:1.8	Vasopressin	1 IU/h
FiO_2_	100 %	Regular insulin	2 IU/h
Ppeak	24 cmH_2_O		
PEEP	8 cmH_2_O		
			

Note: P-ACV: pressure-assist-control ventilation; I/E: inspiration/expiration ratio; FiO_2_: inspiratory fraction of oxygen; Ppeak: peak inspiratory airway pressure; PEEP: positive end-expiratory pressure; IU: international unit.

## 3 Discussion

Hyperkalemia-induced life-threatening arrhythmia is challenging. A previous report of refractory ventricular fibrillation due to hyperkalemia presented the potassium level as high as 8.6 mmol/L. ECMO provided adequate cardiopulmonary support and resuscitation, allowing sufficient time for potassium excretion [5]. Our patient with hyperkalemia demonstrated a potassium level of up to 9.2 mmol/L. This is the highest reported potassium level that induced refractory ventricular fibrillation and was successfully managed with ECMO in adults.

Hyperkalemia management included calcium chloride, sodium bicarbonate, furosemide, insulin, salbutamol, kayexalate, and hemodialysis. Our patient was initially managed for suspected hyperkalemia based on ECG presentations. The inevitable ventricular fibrillation in the patient increased the difficulty in hyperkalemia treatment. Refractory ventricular fibrillation persisted despite emergent cardiopulmonary resuscitation and defibrillation administration. ECPR, a salvage management strategy if traditional CPR fails, is suitable for managing in-hospital cardiac arrest [6]. The refractory and reversible cardiac arrest due to hyperkalemia in our patient is recognized as a good candidate for ECPR. ECMO is helpful for cardiopulmonary life support and potassium elimination through hemofiltration.

Dehydration-induced acute kidney injury and the omission of insulin injections for blood sugar control contributed to hyperkalemia and hyperglycemia with diabetic ketoacidosis in this patient. Hyperosmolality and insulin deficiency were responsible for the transcellular shift of potassium from the cells into the extracellular fluid. After regular insulin injections, adequate hydration, and CVVH treatment during hospitalization, the patient's hyperglycemia with diabetic ketoacidosis subsided, and his renal function gradually improved.

The patient survived prolonged cardiac arrest without neurological deficits. This could be attributed to the following reasons. First, cardiac arrest occurred in the hospital, and high-quality CPR with defibrillation was immediately introduced under the advanced cardiovascular life support algorithm [7]. Cardiopulmonary chest compression can produce 30%–40% of normal cerebral flow during cardiac arrest [8]. Second, hyperkalemia was determined as a reversible cause of cardiac arrest and managed appropriately. Third, ECPR was promptly initiated to provide resuscitation, cardiopulmonary support, and potassium elimination. Fourth, the absence of hypoxemia or hyperoxemia after ROSC helped improve outcomes because both are associated with increased mortality and poor neurological outcomes [9,10].

This patient received no hypothermia management after cardiac arrest following ECMO resuscitation. His initial temperature was lower than 36°C after ROSC. TTM (32°C-36°C for 24 hours) prevents hypoxic–ischemic brain damage after cardiac arrest in comatose patients in previous study [7]. However, recent studies have revealed that TTM did not increase survival after cardiac arrest or improve neurological outcomes [[11], [12], [13]]. So the patient did not receive TTM after ROSC.

## 4 Conclusion

We presented a 45-year-old male patient with refractory ventricular fibrillation secondary to severe hyperkalemia that was successfully managed with ECMO. ECPR was performed because of prolonged ventricular fibrillation despite conventional CPR, defibrillation, and epinephrine administration. The reversible cause of hyperkalemia, early treatment of cardiac arrest, high-quality CPR, and timely application of ECPR resulted in a good prognosis.

## Funding

Not applicable.

## Data availability statement

The original contributions presented in this study are included in the article. Further inquiries can be directed to the corresponding author.

## Additional information

No additional information is available for this study.

## CRediT authorship contribution statement

**Kun-Te Lin:** Writing – review & editing, Writing – original draft, Formal analysis, Data curation. **Fu-Yuan Siao:** Writing – review & editing, Writing – original draft, Conceptualization.

## Declaration of competing interest

The authors declare that they have no known competing financial interests or personal relationships that could have appeared to influence the work reported in this paper.
