# Corrigendum to “Refractory ventricular fibrillation secondary to hyperkalemia resuscitated with extracorporeal membrane oxygenation: A case report” [Heliyon Volume 10, Issue 10, May 30 2024, Article e31178]

**DOI:** 10.1016/j.heliyon.2025.e43316

**Published:** 2025-04-08

**Authors:** Kun-Te Lin, Fu-Yuan Siao

**Affiliations:** a Department of Emergency and Critical Care Medicine, Changhua Christian Hospital, 500, Changhua, Taiwan; b Department of Kinesiology, Health and Leisure, Chienkuo Technology University, 500, Changhua, Taiwan; c Department of Mechanical Engineering, Chung Yuan Christian University, 320, Taoyuan, Taiwan

In this article, the authors omitted a statement in their article regarding informed consent for this publication, which is required by the policies of the journal. The authors confirm that informed consent was obtained from the patient for this publication.

The correct version of the consent statement should be as below.

***Patient consent:***

*The patient provided written informed consent for the publication of their anonymized case details and images*.

The *authors* apologize for the errors.

## Declaration of competing interest

The authors declare that they have no known competing financial interests or personal relationships that could have appeared to influence the work reported in this paper.

